# Ultrasonic Assisted Extraction of Paclitaxel from *Taxus x media* Using Ionic Liquids as Adjuvants: Optimization of the Process by Response Surface Methodology

**DOI:** 10.3390/molecules22091483

**Published:** 2017-09-11

**Authors:** Zhijian Tan, Qiao Li, Chaoyun Wang, Wanlai Zhou, Yuanru Yang, Hongying Wang, Yongjian Yi, Fenfang Li

**Affiliations:** 1 Institute of Bast Fiber Crops and Center of Southern Economic Crops, Chinese Academy of Agricultural Sciences, Changsha 410205, China; aruofly@126.com (W.Z.); yangyuanru@caas.cn (Y.Y.); Cswhy328@126.com (H.W.); ibfcyyj@163.com (Y.Y.); 2 College of Chemistry and Chemical Engineering, Central South University, Changsha 410083, China; liqiao@csu.edu.cn (Q.L.); lfflqq@csu.edu.cn (F.L.)

**Keywords:** paclitaxel, ionic liquids, adjuvants, ultrasonic assisted extraction

## Abstract

(1) Background: Ionic liquids (ILs) are considered “green” solvents and have been widely used in the extraction and separation field in recent years; (2) Methods: In this study, some common ILs and functionalized magnetic ionic liquids (MILs) were used as adjuvants for the solvent extraction of paclitaxel from *Taxus x media* (*T. x media*) using methanol solution. The extraction conditions of methanol concentration, IL type and amount, solid–liquid ratio, extraction temperature, and ultrasonic irradiation time were investigated in single factor experiments. Then, three factors of IL amount, solid–liquid ratio, and ultrasonic irradiation time were optimized by response surface methodology (RSM); (3) Results: The MIL [C_4_MIM]FeCl_3_Br was screened as the optimal adjuvant. Under the optimization conditions of 1.2% IL amount, 1:10.5 solid–liquid ratio, and 30 min ultrasonic irradiation time, the extraction yield reached 0.224 mg/g; and (4) Conclusions: Compared with the conventional solvent extraction, this ultrasonic assisted extraction (UAE) using methanol and MIL as adjuvants can significantly improve the extraction yield, reduce the use of methanol, and shorten the extraction time, which has the potentiality of being used in the extraction of some other important bioactive compounds from natural plant resources.

## 1. Introduction

Paclitaxel (Taxol^®^) is a kind of terpene compound separated from various *Taxus* species. Due to its excellent anticancer effect, paclitaxel is currently the most widely used anticancer drug in clinical practice, including the curing of leukemia, ovarian cancer, breast cancer, Kaposi’s sarcoma, and non-small cell lung cancer [1,2,3]. The demand of paclitaxel is becoming more and more great, however, the content of paclitaxel is very low (about 0.001–0.05%) in dry *Taxus* samples, moreover, the *Taxus* species grow very slowly and are not abundant in the world [4,5]. Thus, how to obtain large amounts of paclitaxel is becoming a serious problem. There are multiple methods used for obtaining paclitaxel, for example, solvent extraction from the crude materials of *Taxus* or the plant cell cultures and the semi-synthesis from the precursors. Among these methods, the extraction of paclitaxel from crude materials using organic solvents is still a non-negligible method for obtaining paclitaxel due to the lower production cost and easier operation [3]. Nevertheless, in order to avoid the destruction of more *Taxus* trees, much work should be done, including the utilization of the branches on *Taxus* and improvement of the extraction efficiency by solvent extraction. It was reported that methanol was the optimal solvent used for the extraction of paclitaxel from *Taxus* among the commonly used organic solvents [6]. However, there are some disadvantages to solvent extraction, such as long extraction time, large quantities of methanol due to the low content of paclitaxel in bark, and the low extraction yield of paclitaxel.

Ionic liquids (ILs) are defined as molten salts which have melting points below or around 100 °C. In recent years, ILs have been considered as one kind of green solvents due to their unique properties, including negligible volatility, low flammability, high thermal and chemical stability, excellent solubility ability, and potential recoverability [7,8,9]. Most importantly, ILs have tunable properties, whose cations and anions can be tailored to obtain various task-specific ILs with different physical and chemical properties [10,11]. Up to now, ILs were widely used in the extraction and separation of bioactive compounds from natural plant resources, such as extraction of caffeine from guaraná seeds [12], isoflavones from *Radix puerariae* [13], chlorogenic from *Boehmerianivea* L. Leaves [14], orientin and vitexin from the flowers of *Trollius chinensis* [15], caffeoylquinic acids from Flos *Lonicerae Japonicae* [16], and so on. Although the ILs were widely used in the extraction of bioactive compounds, their potential threat to the environment and human beings was underestimated, thus, the use of ILs deriving from natural products or some functionalized and recyclable ILs will be an inevitable trend [17,18].

As an effective method deriving from IL-based solid-liquid extraction, the extraction using ILs as adjuvants was also developed in recent years. The extraction efficiency can be obviously improved after addition of small amounts of adjuvants, such as the solvent extraction using organic solvents and aqueous two-phase extraction. For example, K.H. Row used ILs and deep eutectic solvents (DESs) as adjuvants for the extraction of astaxanthin from marine plants using acetone as the extraction solvent, the extraction can be obviously improved by addition of the adjuvants, meanwhile, the DESs had better performance than ILs [19]. J.A.P. Coutinho and co-workers used the ILs as adjuvants to improve the extraction and purification of immunoglobulin G in PEG/salt aqueous two-phase system [20].

In our previous work, it was found that the extraction using magnetic ionic liquids (MILs) as adjuvants to the salting-out extraction system can obtain higher extraction yield compared with no addition of ILs or addition of common ILs [21]. The MILs not only have the same properties to the common ILs, but also exhibit strong response to permanent magnetic field [22]. MILs have more advantages, such as overcoming of the formation of emulsions and the potentiality of being recovered in the presence of an external magnetic field, which has more potential application prospects than those of conventional ILs [23,24]. Due to these unique properties, MILs were used in the extraction and separation field, such as liquid–liquid extraction [25], dispersive liquid–liquid microextraction [26], aqueous two-phase extraction [27], and membrane separation [28]. Thus, the MILs are also feasible for use in the solid–liquid extraction in this work. The common ILs and MILs were used as adjuvants to the ultrasonic assisted extraction (UAE) of paclitaxel using methanol solution. The main factors influencing the extraction were investigated in the single factor experiments, and then the major factors were optimized by response surface methodology (RSM).

## 2. Results and Discussion

### 2.1. Screening of the Optimal IL as Adjuvant

**Figure 1:**
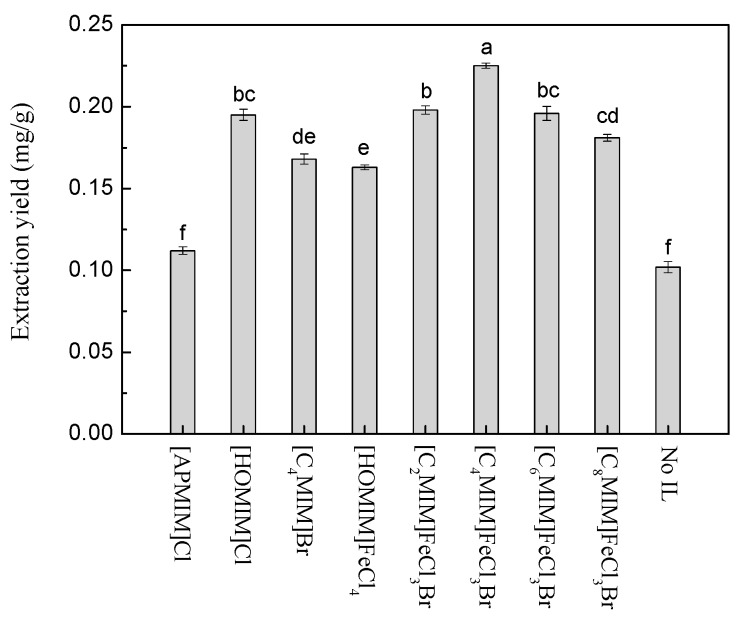
Effect of different ionic liquids (ILs) used as adjuvants to the extraction yields of paclitaxel (Extraction conditions: 0.5 g *T. x media* leaves powder, 60 vol % methanol, 1.0 wt % IL added, 1:10 solid–liquid ratio, 40 °C extraction temperature, and 30 min ultrasonic irradiation time). Different letters in the same series indicate significant difference at *p* < 0.05 level.

In this work, eight imidazolium based ILs were considered as the adjuvants, including three common ILs and five functionalized MILs. It can be seen in Figure 1 that all the extraction yields were obviously improved after addition of ILs compared with the blank (no IL added). The extraction yield using ILs as adjuvants followed this order: No IL added < [APMIM]Cl< [HOMIM]FeCl_4_ ≈ [C_4_MIM]Br < [C_8_MIM]FeCl_3_Br < [HOMIM]Cl ≈ [C_6_MIM]FeCl_3_Br ≈ [C_2_MIM]FeCl_3_Br < [C_4_MIM]FeCl_3_Br. It was reported that the extraction using ILs had been affected by multi-interactions including π-π, n-π, ionic/charge-charge, and hydrogen bonding between the target compounds and the extraction solvents [29]. The co-solvent was formed by methanol and IL; the IL had high polarity and the hydrogen-bond interaction occurred between paclitaxel and ILs, which is beneficial to the extraction. To analyze the results, it can be found that the influencing of cations in ILs was irregular, but the ILs with [FeCl_3_Br]^−1^ as anion had relatively higher extraction yields. The probable reason for this is the stronger π-π interaction that occurred between paclitaxel and [FeCl_3_Br]^−1^ than that which occurred between paclitaxel and other anions. The maximum extraction yield can be obtained using [C_4_MIM]FeCl_3_Br as the adjuvant. Moreover, it was reported that the acidic conditions were beneficial for the stability of paclitaxel [30], which can hydrolyze the 7-xylosyltaxol into paclitaxel leading to the increase of extraction yield [31]. The solution of [C_4_MIM]FeCl_3_Br is acidic, thus, this MIL was chosen for further studies.

### 2.2. Effect of IL Amount 

**Figure 2:**
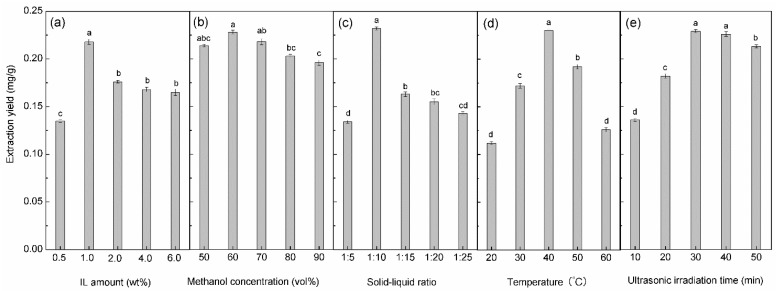
Effect of different factors to the extraction yield of paclitaxel in the single factor experiments. (**a**) the IL amount (0.5 g *T. x media* leaves powder, 60 vol % methanol, 1:10 solid–liquid ratio, 40 °C extraction temperature, and 30 min extraction time); (**b**) Methanol concentration (0.5 g *T. x media* leaves powder, 1.0 wt % IL, 1:10 solid–liquid ratio, 40 °C extraction temperature, and 30 min ultrasonic irradiation time); (**c**) Solid–liquid ratio (0.5 g *T. x media* leaves powder, 60 vol % methanol, 1.0 wt % IL, 40 °C extraction temperature, and 30 min ultrasonic irradiation time); (**d**) Extraction temperature (0.5 g *T. x media* leaves powder, 60 vol % methanol, 1.0 wt % IL, 1:10 solid–liquid ratio, and 30 min ultrasonic irradiation time); (**e**) Ultrasonic irradiation time (*T. x media* leaves powder, 60 vol % methanol, 1.0 wt % IL, 1:10 solid–liquid ratio, and 40 °C extraction temperature). Different letters in the same series indicate significant difference at *p* < 0.05 level.

The effect of the IL amount was investigated at a range of 0.5–6.0 wt %. The results in Figure 2a show that the maximum extraction yield was obtained when 1.0 wt % [C_4_MIM]FeCl_3_Br was added. The probable reason for the further increase of IL resulting in the decrease in extraction yield is that excess IL breaks the hydrogen-bond interaction between IL and methanol, making the polarity of this co-solvent decrease, which is not suitable for the extraction of paclitaxel. Therefore, 1.0 wt % [C_4_MIM]FeCl_3_Br was chosen for further studies.

### 2.3. Effect of Methanol Concentration 

Methanol concentration can be another factor influencing the extraction yield using this co-solvent formed by IL and methanol. Actually, there are many small molecule solvents, such as methanol, ethanol, acetone, dichloromethane, and trichloromethane, which were chosen for the extraction of paclitaxel from *Taxus;* methanol was proved to be the best solvent for the extraction among these solvents. Moreover, it was also reported that a high concentration of methanol (>90%) was more beneficial for the extraction [32,33,34]. The effect of methanol concentration in the range of 50–90 vol % was studied. The results in Figure 2b show that the maximum extraction yield was obtained at the methanol concentration of 60 vol %, then decreased with further increase of methanol concentration. The explanation of the increase of methanol concentration leading to the decrease in extraction yield is similar to that for the effect of IL, the excess of methanol destroys the balance between IL and methanol making this co-solvent not beneficial to the extraction. The introduction of IL to this solvent extraction can obviously reduce the amount of methanol used. Thus, 60 vol % methanol was chosen for further studies.

### 2.4. Effect of Solid–Liquid Ratio

The solid–liquid ratio can affect the extraction to some extent, less solvent leads to inadequate extraction, while excess solvent result in waste and an increase in cost. The solid–liquid ratio (mass of *T. x media* leaves powder to the volume of co-solvent) in the ranges of 1:5, 1:10, 1:15, 1:20, and 1:25 was investigated. The results in Figure 2c shows that the maximum extraction yield was obtained at the solid–liquid ratio of 1:10.Thisindicated that a proper solid–liquid ratio (for example 1:10) was enough for the extraction, while the high solid–liquid ratio (for example 1:5) led to inadequate extraction. The lower solid–liquid ratio (for example below 1:10) made the ultrasonic energy be absorbed and dispersed by a larger volume of solvent, which was disadvantageous for the UAE [35].

### 2.5. Effect of Extraction Temperature

Temperature is an important factor influencing the extraction. In a general way, the higher temperature can accelerate the mass transfer and diffusion, which is beneficial to the extraction. However, excessively high temperature will destroy the stability of some bioactive compounds. In this study, the effect of extraction temperature in the range of 20–60 °C was studied. The results in Figure 2d show that the extraction yield increased with increase in temperature below 40 °C, then decreased with further increment of temperature. The rise in temperature can increase solubility and facilitate paclitaxel diffusion by decreasing viscosity [36,37]. However, the higher temperature also caused degradation of paclitaxel; similar results were reported in other studies about the extraction of paclitaxel [3,36]. Thus, 40 °C was chosen as the optimal temperature.

### 2.6. Effect of Ultrasonic Irradiation Time

It is well known that ultrasonic assisted extraction can shorten the extraction time compared with traditional heat reflux extraction. Thus, the ultrasonic irradiation time of 10–60 min was chosen in this study. It can be seen in Figure 2e that the extraction yield increased with ultrasonic irradiation time before 30 min, then varied little with further increase of irradiation time, thus, the ultrasonic irradiation time of 30 min was chosen.

### 2.7. Optimization of the Extraction by RSM

Due to the shortage of analyses of the interaction between different factors influencing the extraction in single experiments, RSM based on Box–Behnken design (BBD) was used to evaluate the interaction between three major factors and optimize the extraction conditions. Three factors (IL amount, solid–liquid ratio, and ultrasonic irradiation time) and three levels (0.5, 1.0, and 1.5 wt % IL amount; 1:5, 1:10, and 1:15 solid–liquid ratio; and 20, 30, and 40 min ultrasonic irradiation time) were adopted to design the experiments. The list of experimental groups and the obtained results are shown in Table 1.

The ANOVA in Table 2 showed the *p*-value was <0.0001 and Model *F*-value was 154.83, which indicates the regression model and model are significant, respectively. There is a less than 0.01% probability that a “Model *F*-Value” occurs due to statistical noise. The model terms of A, B, A^2^, B^2^, and C^2^ were significant according to their *p* values <0.05. 

The “Lack of Fit *F*-value” of 0.48 implies the “Lack of Fit” is not significant relative to the pure error and that there is a 99.50% chance that a “Lack of Fit F-value” this large could occur due to noise. The regression model was generated by software, which was present in Equation (1):

Y = 0.22 + 0.022A + 7.25 × 10^−3^B + 3.75 × 10^-4^C − 1.5 × 10^-3^AB − 2.5 × 10^−4^AC+ 2.5 × 10^−3^BC − 0.033A^2^ − 0.033B^2^ − 0.015C^2^ (R^2^ = 0.9950) (1)

where Y is the extraction yield of paclitaxel (mg/g), A is the IL amount (wt %), B is the solid–liquid ratio, and C is ultrasonic irradiation time (min). The coefficient of determination (R^2^) is 0.9950, implying that more than 99.50% of the variations in the process efficiency could be explained by the model.

**Figure 3:**
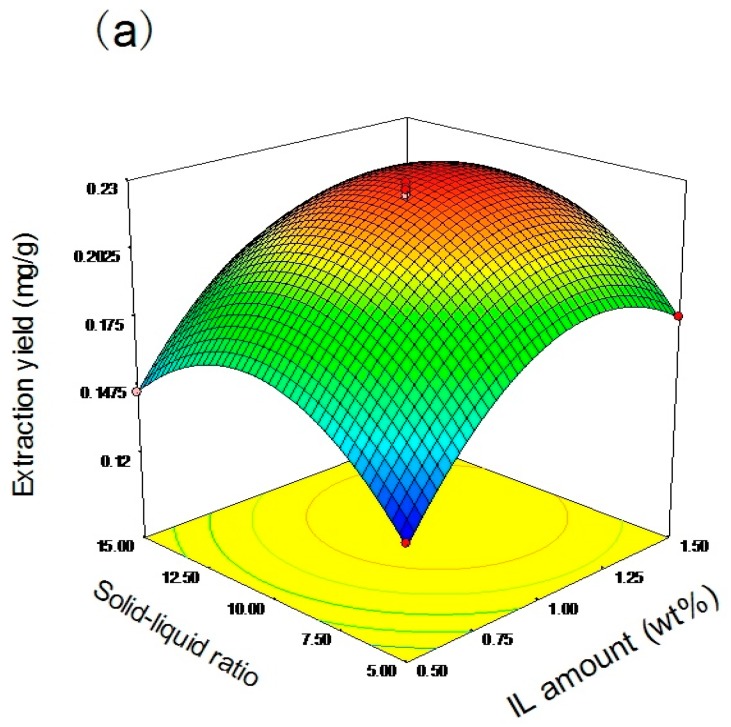

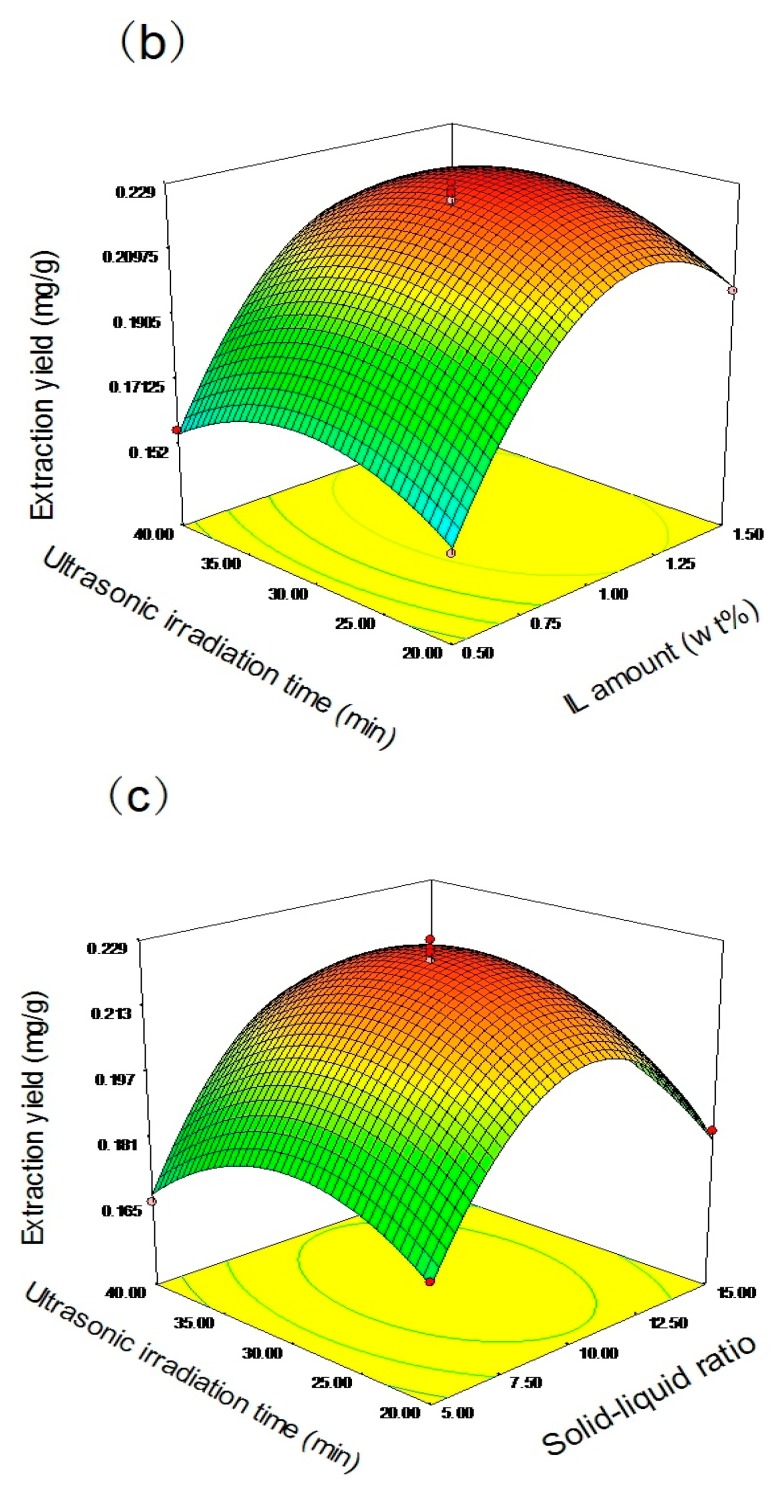
Response surface curves for the extraction of paclitaxel showing interaction between (**a**) IL amount (wt %) and solid–liquid ratio; (**b**) IL amount (wt %) and ultrasonic irradiation time (min), and (**c**) solid–liquid ratio and ultrasonic irradiation time (min).

The response surfaces for the effects of the independent variables on the average extraction yield of paclitaxel (mg/g) are shown in Figure 3. The ordinate shows the extraction yields and the abscissa shows any two variables. The three-dimensional profiles can indicate how any two variables influence the extraction. It can be seen in Figure 3 that all the surfaces are upper convex with a maximum value at the center of the response surface, which approves the rationality of the predicted models [38]. The optimized conditions were obtained based on the quadratic model and are as follows: 1.17% IL amount (*w*/*w*), 1:10.51 solid–liquid ratio, and 30.16 min ultrasonic irradiation time, giving a predicted extraction yield of 0.2292 mg/g. The verification tests were done at the optimized conditions of 1.2% IL amount, 1:10.5 solid–liquid ratio, and 30 min ultrasonic irradiation time. The experimental extraction yield with the average of triplicate runs was 0.224 mg/g, which was close to the predicted value. The results demonstrate that the model is adequate for predicting the expected optimization.

## 3. Materials and Methods

### 3.1. Materials and Reagents

The *Taxus x media* leaves were obtained from Chongqing Beisheng Pharmachem Co. Ltd. (Chongqing, China). The leaves were harvested from the branches of the 20-year-old trees and dried in the drying oven with a constant temperature of 50 °C. Then, the leaves were smashed to powder and sieved to 80 mesh particle size. The ILs [C_2_MIM]Br, [C_4_MIM]Br, [C_6_MIM]Br, [C_8_MIM]Br, [APMIM]Cl, and [HOMIM]Cl were purchased from Shanghai Chengjie Chemical Co. Ltd. (Shanghai, China) with purity >99%. The other MILs of [HOMIM]FeCl_4_, [C_8_MIM]FeCl_3_Br, [C_6_MIM]FeCl_3_Br, [C_2_MIM]FeCl_3_Br, and [C_4_MIM]FeCl_3_Br were synthesized according to the reported procedures by a simple exchange of anions. The structures of all the ILs used in this study are shown in Table 3, and the ILs were characterized by FT-IR spectroscopy and ^1^H-NMR; the results proved that all synthesized ILs were target products. The paclitaxel standard with purity greater than 98% was purchased from Alladin Reagent Co., Ltd. (Shanghai, China). HPLC grade acetonitrile was obtained from TEDIA Company, Inc. (Fairfield, OH, USA). Methanol and ferric chloride of analytical grade were provided by Sinopharm Chemical Reagent Co., Ltd. (Shanghai, China). Other reagents were of analytical grade and used without further treatment. Deionized water was used to prepare the sample solutions.

### 3.2. Solvent Extraction of Paclitaxel

To a tube, a certain amount of *Taxus x media* leaves powder, methanol solution, and IL were added. The tube was placed in an ultrasonic bath (KQ-5200DE, Kunshan Ultrasound Co. Ltd., Kunshan, China) for the ultrasonic assisted extraction of paclitaxel. The operating conditions of the ultrasonic bath were as follow: 200 W electric power, 40 kHz generators frequency, 50 °C operating temperature, and 30 min ultrasonic irradiation time. The extract was centrifuged at 5000× *g* for 10 min. The supernate was separated and diluted for the analysis of paclitaxel by HPLC. The extraction yield of paclitaxel was defined in the following Equation (2):

(2) $$\mathrm{Extraction}\;\mathrm{yield}\;\left(\mathrm{mg}/g\right)\;=\frac{\mathrm{Mass}\;\mathrm{of}\;\mathrm{paclitaxel}\;\mathrm{determined}\;\left(\mathrm{mg}\right)}{\mathrm{Mass}\;\mathrm{of}\;Taxusx\;media\;\mathrm{leaves}\;\mathrm{powder}\;\left(g\right)}$$

### 3.3. HPLC Conditions

**Figure 4:**
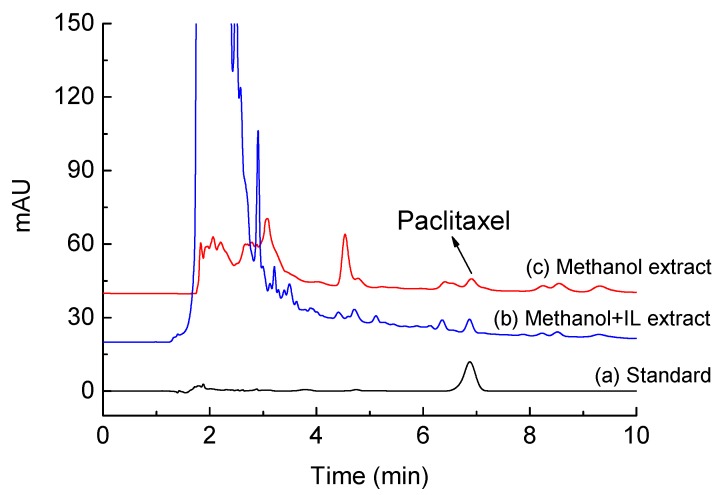
HPLC chromatograms for paclitaxel extracted from *Taxus x media* leaves. (**a**) Standard; (**b**) methanol + IL extract; and (**c**) methanol extract.

A Dionex UltiMate 3000 HPLC system (Dionex, Sunnyvale, CA, USA) was equipped with a pump (model LPG-3400), a variable wavelength UV-Vis detector (model VWD-3400), and a RS column compartment (model TCC-3000). AKromasil 100-5-C_18_ reversed-phase chromatographic column (250 × 4.6 mm i.d., 5 μm) was used to analyze the samples. The mobile phase was acetonitrile-pure water (60:40, *v*/*v*). The flow rate was 1.0 mL/min with isocratic elution. The effluent was monitored at the wavelength of 227 nm and the oven temperature was set at 28 °C. Each sample of 20 μL was injected into the HPLC system. All the samples and mobile phase were filtered through microfiltration membrane (0.45 μm) before analysis. The standard curve for analysis of paclitaxel is obtained in Equation (3):

Y = 0.66204X − 0.16951 (r^2^ = 0.9998) (3)

where Y was the peak area and X was the concentration of standard paclitaxel with the linear range of 5.0–80 μg/mL. The HPLC chromatograms for paclitaxel extracted using methanol with and without addition of IL are shown in Figure 4.

### 3.4. Statistical Analysis

Design Expert 7.0 (DE, Stat-Ease, Inc., Minneapolis, MN, USA) was used to analyze the experimental data and obtain the response models. The values for coefficient of determination (R^2^) and lack of fit were used to judge the suitability of the model. The analysis of variance (ANOVA) was carried out with the comparison of the experimental and predicted values. *p* values <0.05 were considered statistically significant. The optimized conditions for the variables were acquired by statistical analysis. The statistical analysis for screening the ILs and single factor experiments was done using the Duncan’s multiple range test. Each error bar indicates the standard deviation of triplicate tests.

## 4. Conclusions

In this work, the MIL [C_4_MIM]FeCl_3_Br was used as the adjuvant to the ultrasonic assisted extraction of paclitaxel from *T. x media* using methanol solution. The influencing factors of IL amount, methanol concentration, solid–liquid ratio, extraction temperature, and ultrasonic irradiation time in single factor experiments were investigated. Then, the major factors of IL amount, solid–liquid ratio, and ultrasonic irradiation time were optimized by RSM. The extraction yield reached 0.224 mg/g under the optimized conditions of 1.2% IL amount, 1:10.5 solid–liquid ratio, and 30 min ultrasonic irradiation time. Compared with conventional solvent extraction, this ultrasonic assisted extraction using ILs as adjuvants can reduce the usage of methanol, shorten the extraction time, and raise the extraction yield, which has the prospect for application in the extraction of some important bioactive compounds from natural plants.

## Figures and Tables

**Table 1:** Experimental results for the three-factor/three-level Box–Behnken design (BBD).

Runs	Factor A: IL Amount (wt %)	Factor B: Solid–Liquid Ratio	Factor C: Ultrasonic Irradiation Time(min)	Extraction Yield (mg/g)
1	0.5	1:5	30	0.129
2	1.5	1:5	30	0.176
3	0.5	1:15	30	0.145
4	1.5	1:15	30	0.186
5	0.5	1:10	20	0.152
6	1.5	1:10	20	0.198
7	0.5	1:10	40	0.156
8	1.5	1:10	40	0.201
9	1.0	1:5	20	0.172
10	1.0	1:15	20	0.183
11	1.0	1:5	40	0.165
12	1.0	1:15	40	0.186
13	1.0	1:10	30	0.229
14	1.0	1:10	30	0.224
15	1.0	1:10	30	0.219
16	1.0	1:10	30	0.227
17	1.0	1:10	30	0.226

**Table 2:** Analysis of variance (ANOVA) for the quadratic response surface model.

Source	Sum of Squares	Degree of Freedom	Mean Square	*F*-Value	*p*-Value Prob > *F*
Model	1.60 × 10^−2^	9	1.742 × 10^−3^	154.83	<0.0001
A	4.005 × 10^−3^	1	4.005 × 10^−3^	356.01	<0.0001
B	4.205 × 10^−4^	1	4.205 × 10^−4^	37.38	0.0005
C	1.125 × 10^−6^	1	1.125 × 10^−6^	0.10	0.7611
AB	9.00 × 10^−6^	1	9.00 × 10^−6^	0.80	0.4008
AC	2.50 × 10^−7^	1	2.50 × 10^−7^	0.022	0.8857
BC	2.50 × 10^−5^	1	2.50 × 10^−5^	2.22	0.1797
A^2^	4.551 × 10^−3^	1	4.551 × 10^−3^	404.50	<0.0001
B^2^	4.62 × 10^−3^	1	4.62 × 10^−3^	410.67	<0.0001
C^2^	9.953 × 10^−4^	1	9.953 × 10^−4^	88.47	<0.0001
Residual	7.875 × 10^−5^	7	1.125 × 10^−5^		
Lack of Fit	2.075 × 10^−5^	3	6.917 × 10^−6^	0.48	0.7153
Pure Error	5.8 × 10^−5^	4	1.45 × 10^−5^		
Cor Total	1.61 × 10^−2^	16			

**Table 3:** The full names and chemical structures of the ILs used in this study.

Abbreviations of ILs (Full Names)	Cations	Anions
[APMIM]Cl (1-aminopropyl-3-methylimidazolium chloride)	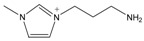	Cl^-^
[C_2_OHMIM]Cl (1-ethoxyl-3-methyl-imidazolium chloride)	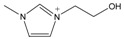	Cl^-^
[C_2_MIM]Br (1-ethyl-3-methyl-imidazolium bromide)	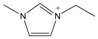	Br^-^
[C_2_OHMIM]FeCl_4_ (1-ethoxyl-3-methyl-imidazolium tetrachloroferrate)	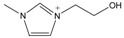	FeCl_4_^-^
[C_2_MIM]FeCl_3_Br (1-ethyl-3-methyl-imidazolium bromotrichloroferrate)	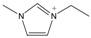	FeCl_3_Br^-^
[C_4_MIM]FeCl_3_Br (1-butyl-3-methyl-imidazolium bromotrichloroferrate)	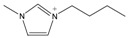	FeCl_3_Br^-^
[C_6_MIM]FeCl_3_Br (1-hexyl-3-methyl-imidazolium bromotrichloroferrate)	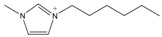	FeCl_3_Br^-^
[C_8_MIM]FeCl_3_Br(1-octyl-3-methyl-imidazolium bromotrichloroferrate)	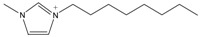	FeCl_3_Br^-^
